# Paraphilic fantasies and behavior in attention deficit/hyperactivity disorder and their association with hypersexuality

**DOI:** 10.1038/s41443-024-00891-w

**Published:** 2024-04-18

**Authors:** Daniel Turner, Priscilla Gregório Hertz, Laura Biedermann, Steffen Barra, Wolfgang Retz

**Affiliations:** 1 Department of Psychiatry and Psychotherapy, University Medical Center Mainz, Mainz, Germany; 2 Institute for Forensic Psychology and Psychiatry, University Medical Center Homburg, Homburg, Germany

**Keywords:** Human behaviour, Diagnosis

## Abstract

Previous research has found some peculiarities in sexual functioning of adults with attention deficit/hyperactivity disorder (ADHD). Using a set of questionnaires that had to be answered online, we assessed the prevalence of paraphilic fantasies and behaviors in a sample of 160 adults with ADHD in comparison to 75 adults without ADHD and evaluated the association between paraphilias and hypersexuality in the ADHD group. Both groups reported high rates of paraphilic fantasies and behaviors. ADHD individuals were more likely to report about very sexually arousing paraphilic fantasies (ADHD: 58.2% vs. non-ADHD: 40.5%; *χ*^*2*^ = 6.323, *p* = 0.01) and behaviors (ADHD: 44.9% vs. non-ADHD: 28.4%; *χ*^*2*^ = 5.774, *p* = 0.02). Furthermore, ADHD individuals reported on average about more very sexually arousing paraphilic behaviors compared to non-ADHD individuals (ADHD: M = 1.28, SD = 0.10 vs. non-ADHD: M = 0.81, SD = 0.09; T = 2.68, *p* < 0.01). Furthermore, in ADHD individuals both very sexually arousing paraphilic interests in masturbation fantasies (*r*(158) = 0.17, *p* = 0.03) and in sexual behaviors (*r*(158) =0.19, *p* = 0.02) showed a significant correlation with hypersexuality. In non-ADHD individuals no such significant correlation could be found. Altogether, it can be concluded that individuals with ADHD seem to be more prone to develop and act out paraphilic sexuality than those without ADHD, however, found differences were rather small. The results of the present study add to the current trend to depathologize paraphilic sexuality in the general as well as in clinical populations.

## Introduction

Attention deficit/hyperactivity disorder (ADHD) is a neurodevelopmental disorder with a worldwide prevalence of about 5 to 7% in childhood and adolescence and a persistence rate of about 50% from childhood to adulthood [1, 2], thus leading to adult prevalence rates between 2 and 3% [3]. Inattention, hyperactivity, and impulsivity constitute the three core symptoms of ADHD, with inattention and impulsivity holding greater relevance for adult ADHD patients than hyperactivity [4]. Furthermore, adult ADHD is frequently accompanied by additional symptoms such as emotional dysregulation, affective lability, low self-esteem, or disorganization and has a high comorbidity rate with other mental disorders such as depressive, anxiety or personality disorders as well as substance abuse [5–8]. ADHD is associated with impaired psychosocial functioning including poor school performance, poor professional success, interpersonal problems, and an increased rate of rule violations [9–11].

Previous research has suggested that ADHD might also go along with peculiarities in sexual functioning and sexual well-being [12, 13]. Thereby, a healthy sexuality is closely related to overall well-being and overall quality of life [14]. This is all the more important since sexuality is frequently neglected during clinical visits, despite patients regularly expressing a desire to talk about their sexuality with their healthcare providers [15, 16]. It has been shown that ADHD is associated with risky sexual behaviors, for example a higher rate of unprotected sex or sex while intoxicated, leading to more sexually transmitted infections and higher rates of teenage pregnancies [17–20].

Hypersexuality usually refers to above average sexual fantasies and behaviors without necessarily implying any psychological strain. With the introduction of the 11^th^ version of the International Classification of Diseases (ICD-11) compulsive sexual behavior disorder (CSBD) was included as an impulse-control disorder [21]. A diagnosis of CSBD is primarily concerned with poorly controlled sexual behaviors. Furthermore, multiple attempts to control or reduce sexual behaviors were unsuccessful and the behavior persists despite negative consequences [21, 22]. A lifetime CSBD prevalence of 4.9% in men and 3.0% in women has been found in the German general population [23]. Depending on the precise definition the prevalence of hypersexuality is usually a bit higher. In a large online study including more than 14,000 individuals, adult ADHD symptoms were associated with problematic pornography use and hypersexuality [24]. Similar findings were recently reported by Gregorio Hertz et al. [13]. In contrast, a recent systematic review and meta-analysis indicated that individuals with ADHD display hypersexual behaviors at a rate similar to that observed in the general population [12, 25]. However, a pooled ADHD prevalence of 20% was found in hypersexual individuals, which is clearly above the ADHD prevalence in the general population [25].

So far, current research lacks an exploration of the associations between ADHD and paraphilias. However, since paraphilias share some symptoms with hypersexuality, it could be hypothesized that they might be more prevalent in individuals with ADHD compared to the general population as well [26, 27]. In the above-mentioned meta-analysis, a pooled ADHD prevalence of 18% was found in individuals with paraphilias, however, this estimate was based on two studies only, underscoring the scarce state of research [25, 28, 29].

The current study aimed at adding to this insufficient state of research concerning paraphilias and ADHD by assessing the prevalence of different paraphilic fantasies and behaviors and their association with hypersexuality in a sample of adults with ADHD.

## Methods

### Data collection and procedure

Data collection was performed between July and October 2021 by means of an online survey. This included among others the German versions of internationally established questionnaires, such as the Self-Report Wender-Reimherr Adult Attention Deficit Disorder Scale (SR-WRAADDS) [30, 31], the Hypersexual Behavior Inventory (HBI-19) [32, 33], and the Questionnaire on Sexual Experiences and Behavior (Q-SEB), which is only available in German [34]. Moreover, we assessed demographic and clinical data, including age, gender, the year of ADHD diagnosis, and specific treatments for ADHD.

The usability and technical functionality of the online questionnaire underwent rigorous testing by three researchers involved in the project at different stages before its official deployment. Adaptive questioning was implemented, meaning that specific items were conditionally displayed based on responses to other items. This approach aimed to streamline the questionnaire, reducing both the number and complexity of questions. The questionnaire comprised items distributed across 16 pages, accompanied by a visible completion bar throughout the survey to enhance participant motivation and, consequently, completion rates. Technical features allowed for a consistency and completeness check before submission, highlighting mandatory questions and partially providing non-response options such as “not applicable” or “rather not say.” Additionally, participants had the ability to review and modify their answers using a Back button, adding a layer of flexibility to the survey experience.

The clinical group, comprising individuals diagnosed with ADHD, was primarily recruited from the ADHD outpatient care center at the Department of Psychiatry and Psychotherapy of the University Medical Center Mainz in Germany. Individuals undergo a comprehensive diagnostic procedure involving questionnaires, clinical interviews, and neuropsychological assessments. Upon completing the diagnostic process, those with a positive diagnosis who were willing to participate in the study were provided with a personal link to a closed online survey via email. The participation in the online survey was entirely voluntary and was in no way connected to the diagnostic process. Recruitment of the control group composed of non-ADHD participants from the general population occurred online without previous initial contact in an open survey through a convenience sampling approach. The study call was posted on social media platforms mostly used to discuss sexual health related topics and was also disseminated through different mailing lists, which were associated with psychology university departments in Germany, to reach out to potential participants. No techniques were used to prevent multiple entries from the same individuals in the open survey.

Before participation, all individuals were informed about the purpose of the survey and that the current study aimed at assessing different sexual behaviors and at comparing these sexual behaviors between individuals with and without an ADHD diagnosis. Furthermore, participants were informed that all information were collected anonymously, that participation was entirely voluntary, that they had to be at least 18 years of age to be allowed to participate, and that they could stop participating by simply closing the window of their internet browser. Before participants could start answering the survey questions, they had to hit a button at the end of the study information on the first page stating that they had read and understood the study information and that they were willing to voluntarily participate in the study. Moreover, participants were provided with an additional incentive consisting of a chance to win a 20-euro voucher to one online retail company. The survey’s electronic platform used, SoSci Survey, automatically captures responses and generates data records, eliminating the need for manual entry into the database.

The study was evaluated positively by the ethical committee of the medical chamber Hamburg in Germany.

### Measures

#### Self-report Wender-Reimherr adult attention deficit disorder scale (SR-WRAADDS) [30, 31]

The SR-WRAADDS consists of 53 questions that assess ADHD symptomatology on ten subscales: attention difficulties, hyperactivity/restlessness, temper, affective lability, emotional over reactivity, disorganization, impulsivity, oppositional symptoms, academic problems, and social attitudes. All items have to be answered on a five-point Likert scale ranging from 1 “does not apply at all to me” to 5 “applies very well to me”. Higher scores on each subscale represent stronger ADHD symptomatology. The German version has yielded good to excellent psychometric properties with a Cronbach’s alpha of 0.95 for the total score and Cronbach’s alphas ranging from 0.70 to 0.87 for the subscales.

#### Hypersexual behavior inventory (HBI-19) [32, 33]

The HBI-19 is a self-report measure of hypersexual behaviors consisting of 19 items grouped to three subscales: (a) attempting to control sexual thoughts, feelings, and behaviors, (b) attempting to cope with unwanted emotions and life stressors, and (c) experiencing undesirable consequences related to problematic sexual behaviors. All items have to be answered on a five-point Likert scale ranging from 1 “never” to 5 “very often”. Higher scores represent more symptomatology and scores equal or above 53 can be considered as clinically relevant levels of hypersexuality. The German version has yielded good to excellent psychometric properties with a Cronbach’s alpha of 0.90 for the total score and Cronbach’s alphas ranging from 0.78 to 0.86 for the subscales.

#### Questionnaire on sexual experiences and behavior (Q-SEB) [34]

Altogether, the Q-SEB consists of 120 items assessing information about sexual socialization, sexual behaviors and different sexual practices including paraphilic fantasies and behaviors. For the purpose of the present study, only items concerning paraphilic fantasies and behaviors were analyzed. All items refer to an observational period of the last 12 months and all items can be answered on a five-point Likert scale ranging from 1 “not at all” to 5 “very” sexually arousing (for example: Exhibitionism: “To what extent do you find it sexually arousing to expose your genitals in public and eventually masturbate in the process?” or Frotteurism: “How sexually arousing do you find rubbing yourself against other people in public (for example in a crowd) or touching them physically?”). All questions had to be answered first in relation to sexual behaviors and second in relation to masturbation fantasies. The following paraphilias can be assessed with the questionnaire: voyeurism, transvestic fetishism, fetishism, sexual masochism, sexual sadism, exhibitionism, frotteurism, and pedophilia. Additionally, one item in the Q-SEB is designed to capture sexual fantasies and behaviors that do involve non-consensual acts, a concept we subsequently refer to as rape in the following sections. The questionnaire is only available in German. However, it has already been used in different previous studies [34].

### Statistical analyses

We calculated the prevalence of each paraphilic interest as a function of masturbation fantasies and sexual behaviors for both the ADHD- and the non-ADHD group separated by gender. Thereby, all participants who answered that they would rate a specific paraphilic interest as at least little sexually arousing (item score of 2) during their masturbation fantasies or sexual behaviors were considered as having this specific paraphilic interest. In order to analyze the prevalence of practically relevant paraphilic interests in masturbation fantasies and sexual behaviors, we analyzed the prevalence of each paraphilic interest by only considering those participants who answered that they viewed a specific paraphilic interest as quite sexually arousing (item score of 4) or as very sexually arousing (item score of 5). Differences in the prevalence rates were calculated between the groups for both female and male gender separately using chi-square tests.

Furthermore, we analyzed the relationship between the number of paraphilic interests in masturbation fantasies and behaviors and hypersexuality by means of spearman correlations in both the ADHD and the non-ADHD group. Due to the limited number of participants, we decided not to segregate the data by gender for further analyses to ensure robustness, as this could have diminished statistical power and compromised the reliability of the findings. Additionally, the prevalence of practically relevant paraphilic masturbation fantasies and sexual behaviors was calculated in the ADHD-group under consideration of hypersexuality, whereby differences between hypersexual and non-hypersexual ADHD participants were examined using chi-square tests. Finally, we calculated a binary logistic regression with presence of any paraphilic sexual fantasy or behavior as outcome criterion and the HBI-19 sum score, the SR-WRAADDS sum score as well as all subscale scores as predictors. The statistical significance value was set on a threshold of *p* < 0.05 for all the statistical analyses. All statistical analyses were performed using SPSS 26 (IBM).

## Results

### Sample

In total, *N* = 344 individuals started answering the online survey. Only individuals who answered all questions relevant for the present study were included in the subsequent data analyses. The drop-out rate did not differ significantly between the ADHD group and the non-ADHD control group (*χ*^*2*^ = 2.34; *p* = 0.13). ADHD participants who were excluded did not score significantly higher on the SR-WRAADDS than ADHD participants who completed the whole questionnaire. Thus, it is unlikely that non-completion of the study varied systematically depending on ADHD symptom severity. Our final sample consisted of *n* = 234 participants (completion rate of 68.02%) of whom *n* = 160 individuals were diagnosed with ADHD (102 women, 51 men, 4 diverse, 3 missing) and *n* = 74 individuals who were not previously diagnosed with ADHD (47 women, 23 men, 3 diverse, 1 missing). As the sample size of individuals who either assigned themselves to the gender “diverse” or did not answer this question (“missing”) was too small, it was not possible to use their data in the gender-specific analyses.

The ADHD group was on average *M* = 37.6 years old (*SD* = 10.6, range 18–63), while the non-ADHD group was on average *M* = 34.0 years old (*SD* = 11.27, range 18–65). The ADHD group was significantly older than the non-ADHD group (*T* = 2.230; *p* = 0.027). Distribution of gender (*χ*^*2*^ = 0.417; *p* = 0.81) did not differ between the ADHD and non-ADHD participants. The ADHD group had a significantly higher SR-WRAADDS sum score than the non-ADHD group (ADHD: *M* = 205.45, *SD* = 30.94; non-ADHD: *M* = 149.82, *SD* = 52.32; *T* = 8.05 *p* > 0.001) and significantly higher scores on all subscales (for more details see Gregório Hertz et al. [16]), indicating, as expected, clearly more intense ADHD symptomatology in the ADHD group.

### Prevalence of paraphilic sexual interests

Participants with ADHD were not more likely to report about any paraphilic fantasies (ADHD: 68.5% vs. non-ADHD: 66.7%; *χ*^*2*^ = 0.058, *p* = 0.81) or any paraphilic behaviors (ADHD: 68.9% vs. non-ADHD: 65.5%; *χ*^*2*^ = 0.233, *p* = 0.63) compared to participants without an ADHD diagnosis. However, it was found that significantly more ADHD individuals reported about practically relevant paraphilic fantasies (ADHD: 58.2% vs. non-ADHD: 40.5%; *χ*^*2*^ = 6.323, *p* = 0.01) and practically relevant paraphilic behaviors (ADHD: 44.9% vs. non-ADHD: 28.4%; *χ*^*2*^ = 5.774, *p* = 0.02). Furthermore, ADHD individuals reported on average about more practically relevant paraphilic behaviors compared to non-ADHD individuals (ADHD: M = 1.28, SD = 0.10 vs. non-ADHD: M = 0.81, SD = 0.09; T = 2.68, *p* < 0.01). All other comparisons concerning the average number of paraphilias were not statistically significant.

The prevalence rates of paraphilic fantasies and behaviors divided by gender are presented in Tables 1 and 2. Regarding any paraphilic interests, non-ADHD women reported significantly more often about frotteuristic masturbation fantasies and sexual behaviors than women with ADHD. Furthermore, more women with ADHD reported about any practically relevant paraphilic fantasies or behaviors than women without ADHD. Moreover, women with ADHD showed a higher rate of practically relevant fetishistic masturbation fantasies and sexual behaviors and a higher rate of masochistic masturbation fantasies than non-ADHD women. In men, the ADHD participants showed a significantly higher rate of practically relevant sadistic sexual behaviors than men without ADHD. No further significant differences were found in men.

**Table 1:** Prevalence of paraphilic fantasies and behaviors in the ADHD- and Non-ADHD group separated by gender.

Paraphilias	Paraphilic fantasies				Paraphilic behaviors			
	Women		Men		Women		Men	
	ADHD *N* = 102	Non-ADHD *N* = 47	ADHD *N* = 51	Non-ADHD *N* = 23	ADHD *N* = 102	Non-ADHD *N* = 47	ADHD N = 51	Non-ADHD *N* = 23
Fetishism	29 (28.4%)	9 (19.1%)	25 (49.0%)	11 (47.8%)	38 (37.3%)	15 (31.9%)	26 (51.0%)	10 (43.5%)
Fetishistic transvestism	8 (7.8%)	5 (10.6%)	9 (17.6%)	5 (21.7%)	9 (8.8%)	5 (10.6%)	6 (11.8%)	4 (17.4%)
Exhibitionism	6 (5.9%)	7 (14.9%)	3 (5.9%)	1 (4.3%)	6 (5.9%)	4 (8.5%)	4 (7.8%)	0 (0.00%)
Voyeurism	43 (42.2%)	16 (34.0%)	34 (66.7%)	13 (56.5%)	35 (34.3%)	11 (23.4%)	23 (45.1%)	12 (52.2%)
Pedophilia with female victims	5 (4.9%)	4 (8.5%)	6 (11.8%)	1 (4.3%)	3 (2.9%)	4 (8.5%)	2 (3.9%)	0 (0.0%)
Pedophilia with male victims	3 (2.9%)	3 (6.4%)	1 (2.0%)	0 (0.0%)	2 (2.0%)	3 (6.4%)	1 (2.0%)	0 (0.0%)
Masochism	67 (65.7%)	31 (66.0%)	21 (41.2%)	10 (43.5%)	68 (66.7%)	33 (70.2%)	25 (49.0%)	12 (52.2%)
Sadism	42 (41.2%)	16 (34.0%)	28 (54.9%)	10 (43.5%)	53 (52.0%)	19 (40.4%)	31 (60.8%)	12 (52.2%)
Frotteurism	**3** (**2.9%)**	**8** (17.0%) **	7 (13.7%)	3 (13.0%)	**4** (**3.9%)**	**6** (12.8%) *	7 (13.7%)	4 (17.4%)
Rape	4 (3.9%)	5 (10.6%)	8 (15.7%)	5 (21.7%)	1 (1.0%)	3 (6.4%)	3 (5.9%)	2 (8.7%)
Any paraphilia	77 (75.5%)	36 (75.0%)	43 (84.3%)	20 (87.0%)	77 (75.5%)	35 (72.9%)	42 (82.4%)	19 (82.6%)

*ADHD* Attention-Deficit/Hyperactivity Disorder.

Chi-square tests comparing frequency of paraphilic fantasies and behaviors between ADHD and non-ADHD participants; **p < 0.01; *p < 0.05.

Statistically significant differences between ADHD and non-ADHD participants are presented in bold.

**Table 2:** Prevalence of practically relevant paraphilic fantasies and behaviors in the ADHD- and Non-ADHD group separated by gender.

Paraphilias	Paraphilic fantasies				Paraphilic behaviors			
	Women		Men		Women		Men	
	ADHD *N* = 102	Non-ADHD *N* = 47	ADHD *N* = 51	Non-ADHD *N* = 23	ADHD *N* = 102	Non-ADHD *N* = 47	ADHD N = 51	Non-ADHD *N* = 23
Fetishism	8 (7.8%)*	0 (0.0%)*	6 (11.8%)	6 (26.1%)	11 (10.8%)*	0 (0.0%)*	8 (15.7%)	4 (17.4%)
Fetishistic transvestism	2 (2.0%)	0 (0.0%)	4 (7.8%)	1 (4.3%)	1 (1.0%)	0 (0.0%)	1 (2.0%)	1 (4.3%)
Exhibitionism	1 (1.0%)	2 (4.3%)	1 (2.0%)	0 (0.0%)	1 (1.0%)	0 (0.0%)	2 (3.9%)	0 (0.0%)
Voyeurism	17 (16.7%)	5 (10.6%)	14 (27.5%)	6 (26.1%)	12 (11.8%)	3 (6.4%)	9 (17.6%)	3 (13.0%)
Pedophilia with female victims	3 (2.9%)	0 (0.0%)	1 (2.0%)	0 (0.0%)	2 (2.0%)	0 (0.0%)	1 (2.0%)	0 (0.0%)
Pedophilia with male victims	2 (2.0%)	0 (0.0%)	0 (0.00%)	0 (0.00%)	1 (1.0%)	0 (0.0%)	0 (0.00%)	0 (0.00%)
Masochism	49 (48.0%)*	**15 (31.9%)**	11 (21.6%)	4 (17.4%)	34 (33.3%)	12 (25.5%)	10 (19.6%)	1 (4.3%)
Sadism	21 (20.6%)	5 (10.6%)	12 (23.5%)	4 (17.4%)	14 (13.7%)	4 (8.5%)	12 (23.5%)*	**1 (4.3%)**
Frotteurism	2 (2.0%)	2 (4.3%)	1 (2.0%)	2 (8.7%)	1 (1.0%)	1 (2.1%)	1 (2.0%)	0 (0.00%)
Rape	1 (1.0%)	1 (2.10%)	5 (9.8%)	1 (4.3%)	1 (1.0%)	0 (0.0%)	1 (4.3%)	1 (4.35%)
Any paraphilia	59 (57.8%)**	**17 (35.4%)**	31 (60.8%)	12 (52.2%)	45 (44.1%)*	**13 (27.1%)**	23 (45.1%)	7 (30.4%)

*ADHD* Attention-Deficit/Hyperactivity Disorder.

Chi-square tests comparing frequency of paraphilic fantasies and behaviors between ADHD and non-ADHD participants; **p < 0.01; *p < 0.05.

Statistically significant differences between ADHD and non-ADHD participants are presented in bold.

### Association between hypersexuality and paraphilic sexual interests

In the ADHD group any paraphilic interests in masturbation fantasies (*r*(158) = 0.13, *p* = 0.1) and in sexual behaviors (*r*(158) = 0.13, *p* = 0.11) were not significantly correlated with hypersexuality. However, both practically relevant paraphilic interests in masturbation fantasies (*r*(158) = 0.17, *p* = 0.03) and in sexual behaviors (*r*(158) =0.19, *p* = 0.02) showed a significant correlation with hypersexuality. In the non-ADHD group only any paraphilic behaviors (*r*[74] = 0.25, *p* = 0.04) were significantly correlated with hypersexuality, while all other correlation coefficients did not reach the predefined level of significance.

Concerning the prevalence rates of practically relevant paraphilic interests as a function of hypersexuality in the ADHD group (see Table 3), individuals with ADHD who were above the HBI-19 cut-off reported significantly more frequently about any paraphilic fantasies, and about exhibitionistic, voyeuristic, pedophilic (female as well as male victims), and rape masturbation fantasies than adults with ADHD below the HBI-19 cut-off. With regard to sexual behaviors, it was found that ADHD individuals above the HBI-19 cut-off reported significantly more often about any paraphilic behaviors, fetishistic behaviors as well as voyeuristic behaviors, while no other significant differences occurred.

**Table 3:** Prevalence of practically relevant paraphilic fantasies and behaviors in the ADHD-group under consideration of the HBI-19 cut-off.

	Paraphilic fantasies		Paraphilic behaviors	
	Hypersexuality *N* = 43	No Hypersexuality *N* = 115	Hypersexuality *N* = 43	No Hypersexuality *N* = 115
Fetishism	6 (14.0%)	10 (8.5%)	10 (23.3%)*	**12 (10.3%)**
Fetishistic transvestism	3 (7.0%)	4 (3.4%)	2 (4.7%)	1 (0.9%)
Exhibitionism	2 (4.7%)*	**0 (0.0%)**	2 (4.7%)	1 (0.9%)
Voyeurism	16 (37.2%)**	16 (13.7%)	13 (30.2%)**	**9 (7.7%)**
Pedophilia with female victims	4 (9.3%)**	**0 (0.0%)**	2 (4.7%)	1 (0.9%)
Pedophilia with male victims	2 (4.7%)*	**0 (0.0%)**	1 (2.3%)	0 (0.0%)
Masochism	12 (27.9%)	50 (42.7%)	13 (30.2%)	33 (28.2%)
Sadism	10 (23.3%)	24 (20.5%)	10 (23.3%)	18 (15.4%)
Frotteurism	2 (4.7%)	1 (0.9%)	1 (2.3%)	1 (0.9%)
Rape	4 (9.3%)*	2 (1.7%)	1 (2.3%)	0 (0.0%)
Any paraphilia	31 (72.1%)*	**61 (53.0%)**	26 (60.5%)*	**45 (39.1%)**

*ADHD* attention-deficit/hyperactivity disorder, *HBI-19* hypersexual Behavior Inventory, cut-off ≥53.

Chi-square tests comparing frequency of paraphilic fantasies and behaviors between those above (hypersexuality) and below (no hypersexuality) HBI-19 cut-off; **p < 0.01; *p < 0.05.

Statistically significant differences are presented in bold.

Finally, the binary logistic regression showed that in adults with ADHD only the HBI-19 sum score significantly predicted the presence of any practically relevant paraphilic masturbation fantasies (see Table 4). Thereby, it was possible to explain 21.1% of the variance of paraphilic masturbation fantasies. Concerning any practically relevant paraphilic sexual behaviors, again the HBI-19 total score was a significant predictor, together with the SR-WRAADDS subscale temper (see Table 4). Both variables explained 23.5% of the variance of paraphilic sexual behaviors.

**Table 4:** Predicting clinically relevant paraphilic masturbation fantasies and sexual behaviors with hypersexuality (HBI-19) and ADHD symptoms (SR-WRAADDS) by means of Binary Logistic Regression.

	B	SE	Exp(B)	Wald	Sig.	95% CI	
						Lower	Upper
Outcome: Masturbation fantasies							
Constant	−1.296	0.453		8.172	0.004		
HBI sum score	0.042	0.011	1.04	14.28	<0.001	1.02	1.07
Outcome: Sexual behaviors							
Constant	−2.603	0.696	0.074	14.00	< 0.001		
HBI sum score	0.03	0.010	1.03	8.96	0.003	1.01	1.05
SR-WRAADDS temper	0.120	0.056	1.13	4.51	0.034	1.01	1.26

*B* beta coefficient, *SE* standard Error, *Exp(b)* exponentiated Beta coefficient, *Sig* significance level, *CI* confidence interval, *ADHD* attention deficit/hyperactivity disorder, *HBI-19* hypersexual behavior inventory, *SR-WRAADDS* Self-Report Wender-Reimherr Adult Attention Deficit Disorder Scale.

## Discussion

The present study is among the first studies to assess the prevalence of different paraphilic sexual interests in adults with ADHD. We found quite considerable numbers of paraphilic masturbation fantasies and paraphilic sexual behaviors in adults with ADHD as well as in non-ADHD adults. In both groups the most frequent paraphilias were fetishistic, voyeuristic and sadomasochistic fantasies and behaviors. Even though the results should be interpreted cautiously because of the rather small sample size and the overrepresentation of women in both groups, these findings are in line with previous studies assessing general population samples that have also found prevalence rates of up to 60% for single paraphilias and that have found voyeurism, fetishism, and masochism to be among the paraphilias reported most frequently [35–37].

Previous studies have suggested that higher degrees of impulsivity are associated with paraphilic sexual interests, which led to the hypothesis that individuals with ADHD might be even more prone to report about paraphilic sexual interests because impulsivity is one of the core symptoms of ADHD [38, 39]. In line, more individuals with ADHD reported about at least one practically relevant paraphilia and ADHD individuals showed on average more practically relevant paraphilic behaviors. However, concerning single paraphilias, with a few exceptions, no considerable differences were found between the two groups. Altogether, these results are consistent with the only other previous study we could identify that has assessed the frequency of different paraphilias in individuals with ADHD and that also found virtually no differences between adults with and without ADHD [40]. The only exceptions were higher rates of frotteuristic fantasies and behaviors among non-ADHD women. It could be possible that due to tactile hypersensitivity, especially in women with ADHD, frotteurism, which is defined as rubbing against a non-consenting person, might be less sexually arousing because tactile stimuli might be perceived as being too intense and thus unpleasant [41]. Speaking against this suggestion, women with ADHD also reported about more practically relevant masochistic sexual fantasies and an increased sensitivity to pain has also been found in ADHD individuals and should thus be perceived as unpleasant as well [42]. Furthermore, individuals engaging in masochistic behaviors rather exhibit pain hyposensitivity, however, it was suggested that this hyposensitivity is the result of the frequent engagement in masochistic behaviors [43]. Since there are probably much less opportunities to engage in frotteuristic behaviors than in masochistic behaviors, this could be a possible explanation for this counterintuitive finding.

Studies on the association of hypersexuality and paraphilias are scarce although both constructs share some characteristics, again especially an increased impulsivity [24, 39, 44]. Since a few studies have found an association between hypersexuality and ADHD, it could be hypothesized that adults with ADHD showing signs of hypersexuality could also report about a higher rate of paraphilic fantasies and behaviors [28, 45]. Indeed, we could show that hypersexuality was positively associated with practically relevant paraphilic fantasies and behaviors in adults with ADHD. Interestingly, this association was not found in the non-ADHD group. This might be explained by the higher impulsive traits in ADHD. However, in the logistic regression not impulsivity but temper was the only ADHD associated symptom that significantly predicted the presence of any paraphilic sexual behaviors in the ADHD group. Similarly, in a previous study, we found that hypersexuality is related to symptoms of emotional dysregulation in adults with ADHD [13]. It has been suggested that sexual behaviors are used by some individuals to relieve negative affect and to cope with affective symptoms, which might be the reason why temper as a specific feature of emotional dysregulation is also related to paraphilic behaviors.

Although the results of the current study clearly add to the scarce state of research concerning sexuality related issues in adults with ADHD, they should be interpreted cautiously due to the exploratory nature of the study and the quite small sample size.

The primary limitation of the present study revolves around a potential sampling bias apparent in the composition of the control group. Specifically, the ADHD subjects constitute a clinically identified group, in contrast to the comparison group, which was recruited solely through social media and mailing lists. To enhance the robustness of the results, employing established panels for the comparison group could have yielded a more thoroughly characterized cohort, especially in terms of socio-demographic characteristics and other pertinent aspects, facilitating a more precise comparison between the samples. However, this recruitment method was adopted for reasons related to accessibility and outreach. In the ADHD group, a possible bias arises from potential co-morbidities, such as antisocial traits and co-occurring autism among many others, which we did not assess. These co-existing conditions may influence paraphilic interests and behaviors, potentially serving as confounding factors in our analysis. For instance, co-occurrence of ADHD with antisocial traits may contribute to impulsive and risky behaviors and delinquency, potentially exacerbating the manifestation of paraphilic interests [11]. Furthermore, co-occurring autism spectrum disorder (ASD) in individuals with ADHD could also amplify the expression of hypersexual behavior and paraphilic interests [46]. By addressing these potential confounders, along with other comorbidities such as depression, social anxiety, and substance disorders, a more nuanced understanding of the relationship between ADHD and paraphilic interests could be achieved. Furthermore, there is a notable overrepresentation of women in the present sample, despite the well-documented higher prevalence of paraphilias and hypersexuality among men. This introduces a potential threat to external validity and raises concerns about confounding effects, particularly if women in the sample are, for instance, more open about their sexuality. Furthermore, conducting subgroup analyses based on gender within two substantially differently sized samples may inadvertently provide more robust statistical power for the overrepresented group, in this case, women, potentially overshadowing crucial trends or effects within the male subgroup. Nevertheless, this study contributes to this specific research area, since paraphilias and hypersexuality in females have been overlooked in research, leading to misconceptions about their occurrence among women. Traditionally, the limited attention given to female samples in studies may have reinforced the misbelief that women with abnormally increased sexual drive or nonnormative sexual interests, for instance pedophilia, are rare or do not exist [47, 48], which is not the case.

Additionally, since we had mentioned in our study call that we would assess different sexual behaviors, it is all the more possible that individuals with a primarily high interest in sexuality-related topics were more likely to participate, possibly leading to a general overestimation of the prevalence of paraphilic fantasies and behaviors. However, all studies assessing sexuality-related constructs have to deal with this problem. Further, we did not specifically assess whether or not ADHD individuals were currently treated with pharmacological agents although especially stimulants, which are frequently prescribed in ADHD, could have an influence on sexual fantasies and behaviors, something that should thus be considered in future research. Moreover, we did not evaluate whether or not paraphilic fantasies and behaviors were generally perceived as positive or negative by our participants and therefore, it is not possible to draw any conclusions on whether paraphilic disorders are a relevant issue for ADHD adults and should thus be considered during clinical visits. This would be an important topic for future research because sexual issues, despite their importance for mental health, are frequently neglected during clinical visits mainly due to the insufficient knowledge of health care providers [16, 49, 50].

Altogether we could show that adults with ADHD report about paraphilic fantasies and behaviors only a little bit more often than adults without ADHD. The high prevalence of paraphilic fantasies and behaviors found in the current study clearly underscores the current trend to depathologize paraphilias in the general as well as in clinical populations and to move away from the view that paraphilias should be considered as “unusual” sexual interests [51].

## Data Availability

The dataset analyzed during the current study is available from the corresponding author on reasonable request.
